# Integrated metabolic and transcriptional analysis reveals the role of carotenoid cleavage dioxygenase 4 (IbCCD4) in carotenoid accumulation in sweetpotato tuberous roots

**DOI:** 10.1186/s13068-023-02299-y

**Published:** 2023-03-14

**Authors:** Jie Zhang, Liheng He, Jingjing Dong, Cailiang Zhao, Yujie Wang, Ruimin Tang, Wenbin Wang, Zhixian Ji, Qinghe Cao, Hong’e Xie, Zongxin Wu, Runzhi Li, Ling Yuan, Xiaoyun Jia

**Affiliations:** 1 College of Agriculture, Shanxi Agricultural University, Jinzhong, China; 2 Department of Life Sciences, Changzhi University, Changzhi, China; 3 State Key Laboratory of Cotton Biology, Henan Joint International Laboratory for Crop Multi-Omics Research, School of Life Sciences, Henan University, Kaifeng, China; 4 College of Life Sciences, Shanxi Agricultural University, Jinzhong, China; 5 Institute of Crop and Nuclear Technology Utilization, Zhejiang Academy of Agricultural Sciences, Hangzhou, China; 6 Xuzhou Sweetpotato Research Center, Xuzhou Institute of Agricultural Sciences, Key Laboratory of Sweetpotato Biology and Genetic Breeding, Ministry of Agriculture, Xuzhou, China; 7 Institute of Cotton Research, Shanxi Agricultural University, Yuncheng, China; 8 Department of Plant and Soil Sciences, Kentucky Tobacco Research & Development Center, University of Kentucky, Lexington, USA

**Keywords:** *Ipomoea batatas*, Gene expression, Carotenoids, Tuberous root development, Carotenoid cleavage dioxygenase 4(IbCCD4)

## Abstract

**Background:**

Plant carotenoids are essential for human health, having wide uses in dietary supplements, food colorants, animal feed additives, and cosmetics. With the increasing demand for natural carotenoids, plant carotenoids have gained great interest in both academic and industry research worldwide. Orange-fleshed sweetpotato (*Ipomoea batatas*) enriched with carotenoids is an ideal feedstock for producing natural carotenoids. However, limited information is available regarding the molecular mechanism responsible for carotenoid metabolism in sweetpotato tuberous roots.

**Results:**

In this study, metabolic profiling of carotenoids and gene expression analysis were conducted at six tuberous root developmental stages of three sweetpotato varieties with different flesh colors. The correlations between the expression of carotenoid metabolic genes and carotenoid levels suggested that the carotenoid cleavage dioxygenase 4 (*IbCCD4*) and 9-cis-epoxycarotenoid cleavage dioxygenases 3 (*IbNCED3*) play important roles in the regulation of carotenoid contents in sweetpotato. Transgenic experiments confirmed that the total carotenoid content decreased in the tuberous roots of *IbCCD4-*overexpressing sweetpotato. In addition, *IbCCD4* may be regulated by two stress-related transcription factors, IbWRKY20 and IbCBF2, implying that the carotenoid accumulation in sweeetpotato is possibly fine-tuned in responses to stress signals.

**Conclusions:**

A set of key genes were revealed to be responsible for carotenoid accumulation in sweetpotato, with *IbCCD4* acts as a crucial player. Our findings provided new insights into carotenoid metabolism in sweetpotato tuberous roots and insinuated *IbCCD4* to be a target gene in the development of new sweetpotato varieties with high carotenoid production.

**Supplementary Information:**

The online version contains supplementary material available at 10.1186/s13068-023-02299-y.

## Background

Plant carotenoids exist not only in flowers and fruits but also in other organs or tissues, such as roots, leaves, and seeds. Carotenoids can be classified into two groups: carotenes (including α-carotene, β-carotene, phytoene, and lycopene) and xanthophylls (such as lutein, cryptoxanthin, and zeaxanthin). Some carotenoids exert beneficial effects on the delay and treatment of cardiovascular disease, cataracts, and night blindness [1, 2]. β-carotene, the precursor of vitamin A, cannot be synthesized by most animals, including humans [3]. Vitamin A deficiency, a serious health concern in developing countries, can result in night blindness and premature death [4]. Carotenoids can be obtained via chemical synthesis or extraction from natural sources. At present, 80–90% of carotenoids in the market are met by chemical synthesis. However, naturally-produced carotenoids are preferable, partly due to the consumer concern of the synthetic compounds [5].

Sweetpotato (*Ipomoea batatas* [L.] Lam.) is an important food and feed crop, rich in many health-promoting components, such as vitamins, minerals, dietary fiber, anthocyanins, and carotenoids [6, 7]. The flesh colors of sweetpotato cultivars include white, yellow, orange, and purple [3]. Orange-fleshed sweetpotato (OFSP) has been highlighted as a health crop to alleviate vitamin A deficiency owning to its high content of β-carotene. In addition, high carotenoid contents in sweetpotato are associated with defending against abiotic stresses such as salt stress, osmotic stress, and drought stress [8–11].

**Fig. 1:**
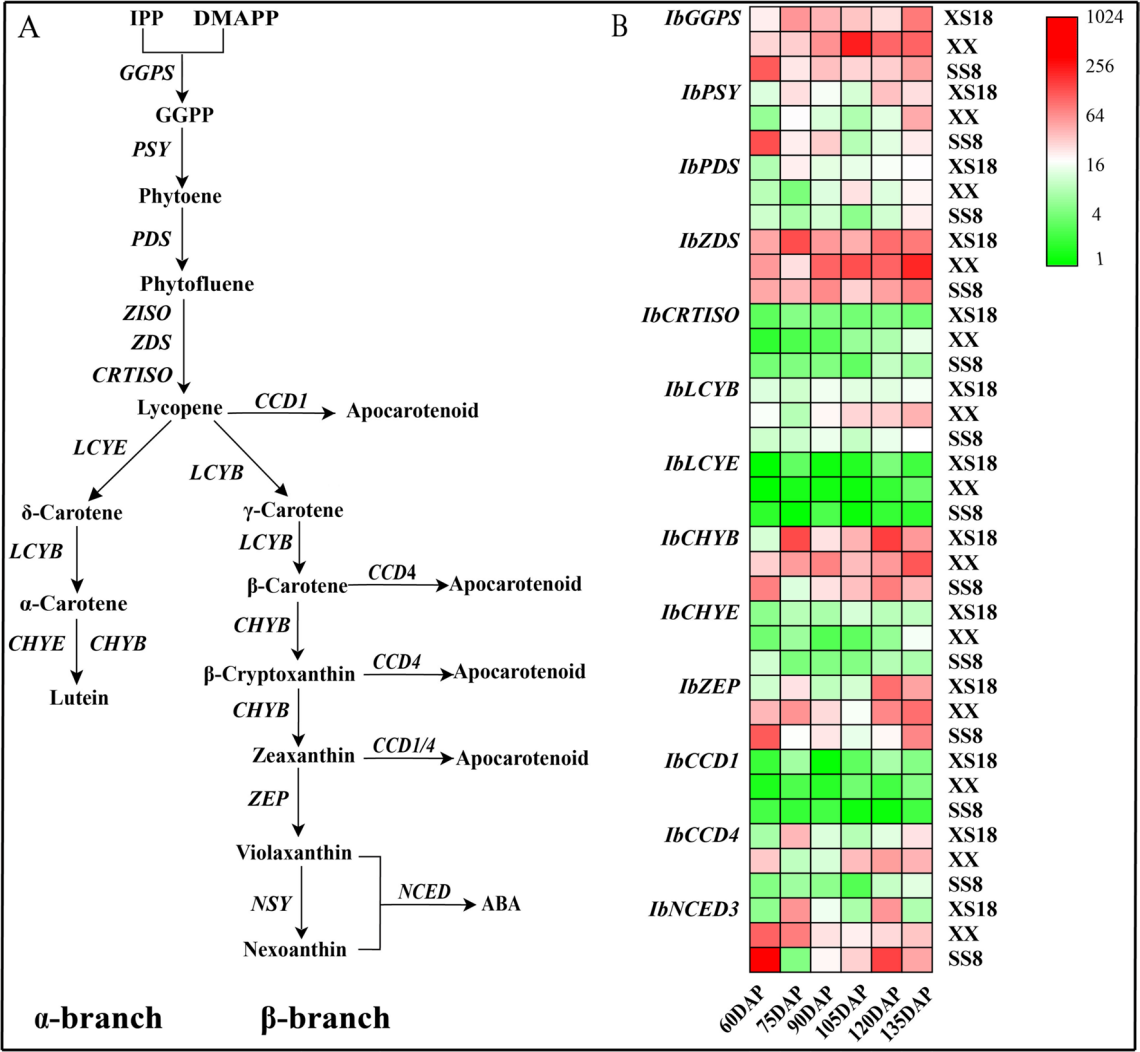
Plant carotenoid metabolic pathway and the expression profiling of thirteen genes by RT-qPCR. **A** The enzymes/genes in the pathway are as follows: GGPS, geranylgeranyl diphosphate synthase; PSY, phytoene synthase; PDS, phytoene desaturase; ZISO, ζ-carotene isomerase; ZDS, ζ-carotene desaturase; CRTISO, carotene isomerase; LCYB, lycopene β-cyclase; LCYE, lycopene Ɛ-cyclase; CHYB, β-carotene hydroxylase; CHYE, Ɛ-carotene hydroxylase; ZEP, zeaxanthin epoxidase; NSY, neoxanthin synthase; CCD, carotenoid cleavage dioxygenase; NCED, 9-cis-epoxycarotenoid dioxygenase. The metabolites are as follows: IPP, isopentenyl diphosphate; DMAPP, dimethylallyl diphosphate; GGPP, geranylgeranyl diphosphate; Phytoene; phytofluene; lycopene; α-carotene; β-carotene; δ-carotene; γ-carotene; lutein; β-cryptoxanthin; zeaxanthin; antheraxanthin; violaxanthin; neoxanthin; ABA (abscisic acid); apocarotenoids (figure is modified from the reports [22–24]). **B** Expression profiles of carotenoid metabolic genes (*IbGGPS, IbPSY, IbPDS, IbZDS, IbCRTISO, IbLCYB, IbLCYE, IbCHYB, IbCHYE, IbZEP, IbCCD1, IbCCD4,* and *IbNCED3*) in the three sweetpotato varieties with different flesh colors during six tuberous root developmental stages [60, 75, 90, 105, 120, and 135 days after planting (DAP)]. *IbActin* was used as an internal control. *IbCCD1* expression level at 60 DAP in WFSP cv. XS18 was used for calibration. Actual relative expression levels are listed in Additional file 5: Table S4

The carotenoid metabolic pathway in higher plants has been well elucidated as illustrated in Fig. 1a [12–14]. Geranylgeranyl pyrophosphate synthase (GGPS) catalyzes the first step of carotenoid biosynthesis to produce geranylgeranyl diphosphate (GGPP) from isopentenyl diphosphate (IPP) and dimethylallyl diphosphate (DMAPP). Two GGPP molecules are then condensed into colorless phytoene by phytoene synthase (PSY), which is a rate-limiting step [15]. Subsequently, phytoene is converted to red lycopene by phytoene desaturase (PDS), ξ-carotene desaturase (ZDS), carotenoid isomerase (CRTISO), and carotene isomerase (ZISO). The next step is the cyclization of lycopene, which is the branching point of the pathway. Lycopene is catalytically converted to α-carotene by lycopene β-cyclase (LCYB) and lycopene Ɛ-cyclase (LCYE), or to β-carotene by only LCYB. Lutein is generated from α-carotene by β-ring hydroxylase (CHYB) and Ɛ-ring hydroxylase (CHYE). β-cryptoxanthin and zeaxanthin are produced from β-carotene by CHYB, and zeaxanthin is then converted to violaxanthin by zeaxanthin epoxidase (ZEP), with antheraxanthin as an intermediate. Neoxanthin synthase (NSY) converts violaxanthin into neoxanthin. The carotenoid cleavage dioxygenases (CCDs) cleave carotenoids into apocarotenoids (Fig. 1a), which possess the known physiological functions [16]. Two CCD subfamilies have been described, grouping as CCDs (CCD1, CCD4, CCD7, and CCD8) and 9-cis-epoxycarotenoid cleavage dioxygenases (NCED2, NCED3, NCED5, NCED6, and NCED9) [16, 17]. CCDs cleave various carotenoids into apocarotenoids, including monochromatic lactone (SL) and volatile aromatic compounds. NCEDs mainly cleave 9-*cis*-epoxycarotenoids to produce the plant hormone abscisic acid (ABA) [17]. CCD4 has been reported to play a role in the degradation of carotenoids in flowers and fruits of apricot [18], *Chrysanthemum* [19], and peaches [20], as well as potato tubers [21].

A previous study showed a direct correlation between flesh color and carotenoid content in yellow- or orange-fleshed sweetpotato [25]. However, the mechanisms governing carotenoid accumulation in the tuberous roots of sweetpotatoes with different flesh colors remained unclear. The accumulations of specialized metabolites such as carotenoids are mainly controlled at the gene transcription level. In kiwifruit, β-carotene content positively correlates to the expression of *AdLCYB* [26]. High transcript levels of *ClPSY* and *ClGGPS* lead to high carotenoids in red-fleshed watermelon during 18–30 days after pollination [27]. Previous studies on sweetpotato carotenoid metabolism have mainly focused on the carotenoid biosynthetic genes (e.g. *IbGGPS, IbZDS, IbLCYE, IbLCYB, IbCHYB,* and *IbZEP*) [28, 29]. Thus, less is known on the roles of genes controlling carotenoid catabolism or degradation. Carotenoids play significant roles in plant stress responses. Overexpression of *IbGGPS* increases the total carotenoid content and tolerance to osmotic stress in *Arabidopsis thaliana* [11]. The down-regulation of *IbLCYB, IbCHYB,* and *IbLCYE* increases β-carotene content and tolerance to abiotic stress in transgenic sweetpotato calli [8–10]. The *IbZDS*-overexpressing sweetpotato with increased β-carotene and lutein exhibits enhanced salt tolerance [30].

Combined transcriptomic and metabolomic analyses have been used to identify genes associated with carotenoid content in the tuberous roots of sweetpotato [31, 32]. A total of 15 transcription factors were identified to be possibly involved in the regulation of carotenoid accumulation [31]. The enzymes PSY, CHYB, ZEP, NCED3, ABA2 (xanthoxin dehydrogenase), and CYP707A (abscisic acid 8′-hydroxylase) are closely associated with carotenoid biosynthesis [32]. However, no systematic profiling of temporal carotenoid accumulation and gene expression during sweetpotato tuberous root development have been reported, and the sweetpotato carotenoid catabolic genes (e.g., *CCDs* and *NCEDs*) have not been functionally characterized through transgenic experiments.

In this study, high-performance liquid chromatography-tandem mass spectrometry (UHPLC-APCI-MS/MS) was used to quantify carotenoid accumulation during six tuberous root developmental stages of sweetpotatoes with three flesh colors. The resulting data were combined with quantitative real-time polymerase chain reaction (RT-qPCR) analysis of thirteen genes involved in the carotenoid metabolic pathway to establish the correlation between carotenoid biosynthesis and gene expression in tuberous roots of sweetpotato. The *IbCCD4* expression was positively correlated with transcriptions of *IbGGPS*, *IbPDS*, *IbZDS*, *IbLCYB*, and *IbLCHYB*. The expression level of *IbCCD4* was higher in the white- or yellow-fleshed sweetpotato varieties than in the orange-fleshed variety. We subsequently generated *IbCCD4-*overexpressing sweetpotato lines, which showed decreased carotenoid accumulation in tuberous roots. We also identified two transcription factors that transactivate the *IbCCD4* promoter. These results advance our understanding of carotenoid metabolism in the tuberous roots of sweetpotato, establishing *IbCCD4* as a target gene for bioengineering of high-carotenoid sweetpotato using genome editing technology.

## Results

### Differential accumulation of carotenoids in the tuberous roots of three sweetpotato cultivars

**Fig. 2:**
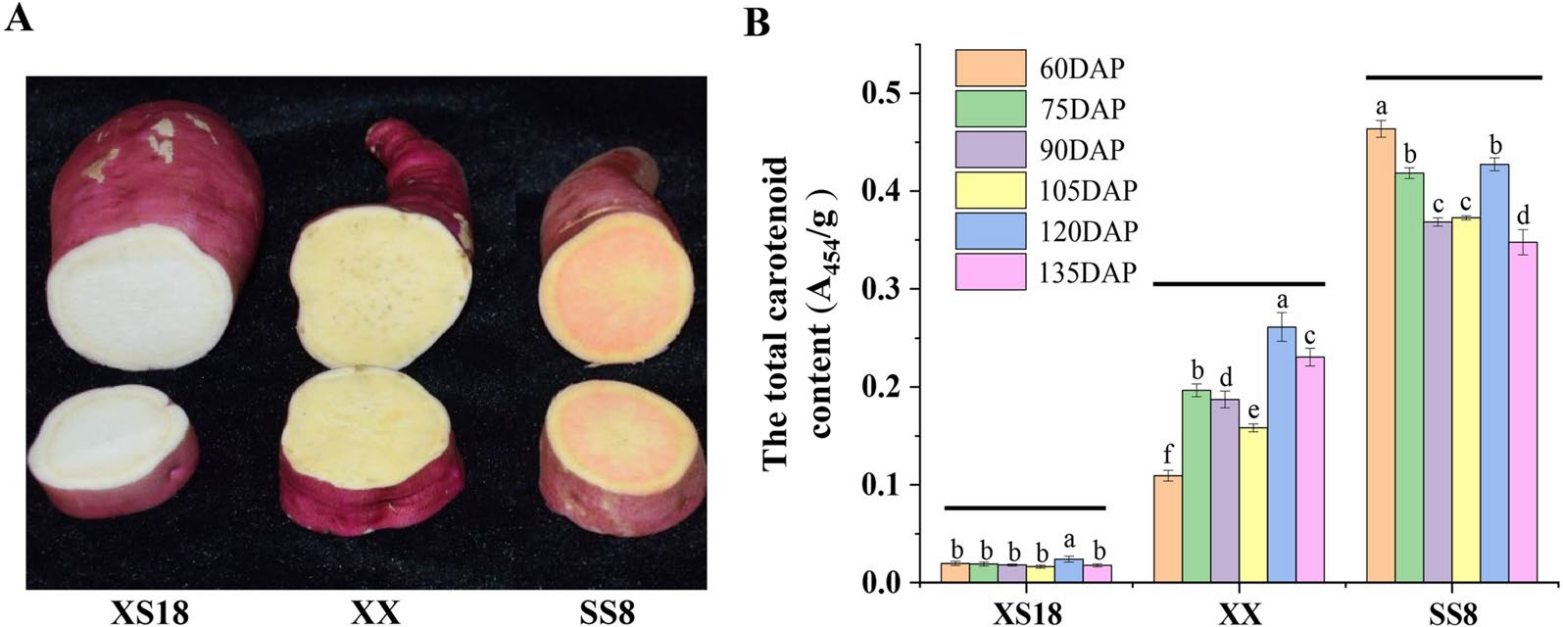
Phenotype of three sweetpotato varieties and dynamic profiles of the total carotenoid content. **A** The color phenotype of tuberous roots of white-fleshed “XS18,” yellow-fleshed “XX,” and orange-fleshed “SS8” at 120 DAP. **B** Total carotenoid contents of XS18, XX, and SS8 at six developmental stages. All data are the means of three biological replicates. Statistically significant differences (*p* < 0.05) are indicated with different letters for the same cultivar

Three sweetpotato varieties with different flesh colors (the white-fleshed XS18, yellow-fleshed XX, and orange-fleshed SS8) were used to quantify the temporal accumulation of carotenoids (Fig. 2a). The carotenoid contents of the three cultivars varied greatly, ranging from 0.016 to 0.464 A_454_/g, during six tuberous root developmental stages (60–135 days after planting or DAP, Fig. 2b). The highest amount of carotenoids were observed in SS8 (0.348–0.464 A_454_/g), followed by XX (0.109–0.261 A_454_/g). Only small amounts of carotenoids (0.016–0.024 A_454_/g) were detected in XS18 (Additional file 5: Table S2). Moreover, the total carotenoid content was well correlated with the color phenotypes of the sweetpotato tuberous roots (Fig. 2a).

The trends of carotenoid accumulation during the six tuber developmental stages differed significantly among the three sweetpotato varieties with different flesh colors (Fig. 2b). The total carotenoid content was the highest at 60 DAP in SS8, and then decreased gradually except for an intermittent increase at 120 DAP. In contrast, the total carotenoid content of XX increased with tuberous root bulking, with the highest peak occurring at 120 DAP. The total carotenoid content remained low during tuberous root development in XS18 (Fig. 2b).

**Fig. 3:**
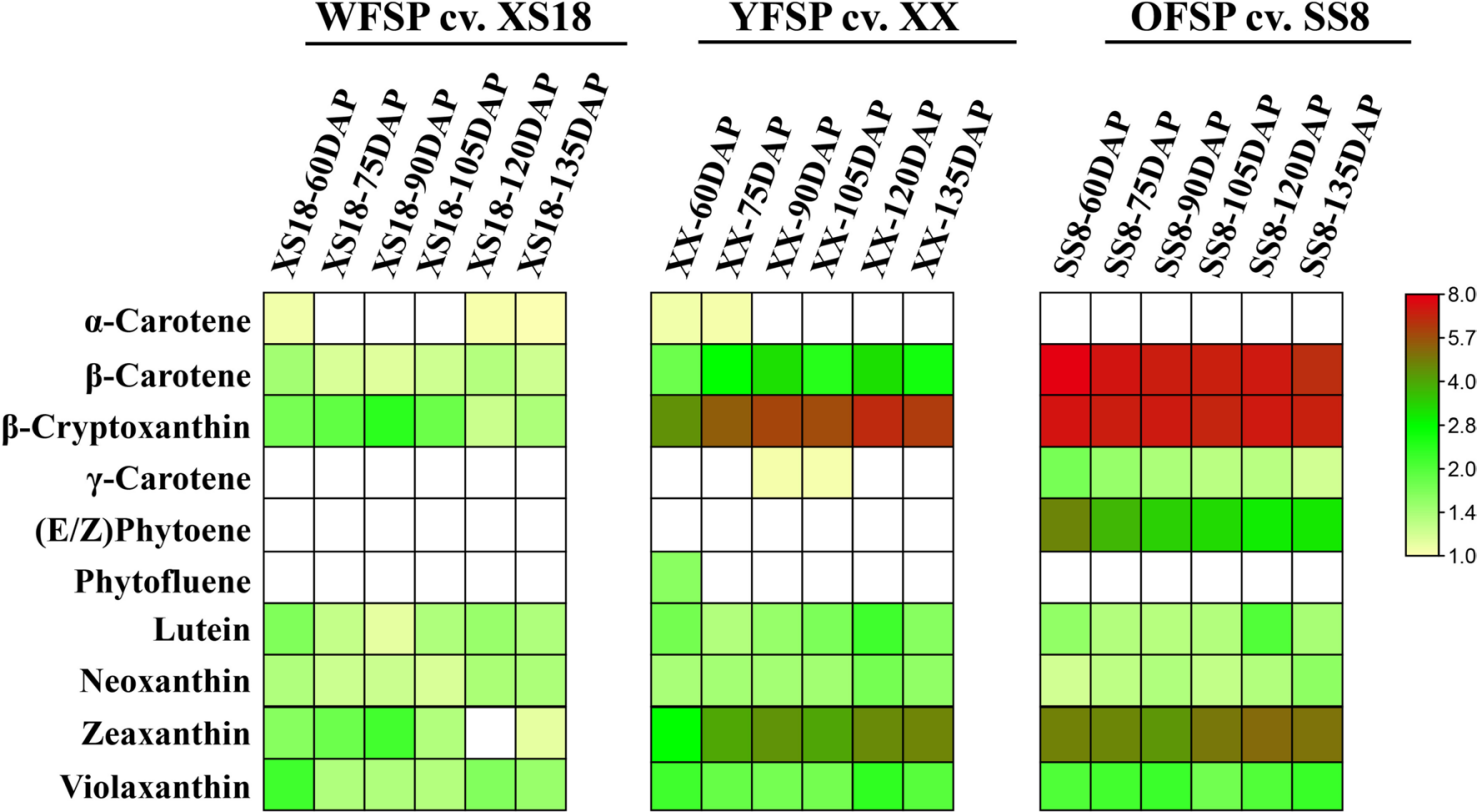
Quantification of the carotenoids in the flesh of three sweetpotato varieties at six tuberous root developmental stages (60, 75, 90, 105, 120, and 135 DAP) (*n* = 3). The results are expressed as μg/g DW. The color intensity scale is presented on the right side of the figure. The actual contents of the carotenoids are listed in Additional file 5: Table S3. “WFSP” is white-fleshed sweetpotato; “YFSP” is yellow-fleshed sweetpotato; and “OFSP” is orange-fleshed sweetpotato

To accurately measure the carotenoid compositions and contents in the tuberous roots of sweetpotato, qualitative and quantitative metabolomic assessments of carotenoids were conducted using UHPLC-APCI-MS/MS. A total of 10 carotenoids were detected in the 18 samples (from six tuberous root bulking stages of the three sweetpotato varieties). They include (E/Z)-phytoene, phytofluene, α-carotene, β-carotene, γ-carotene, β-cryptoxanthin, zeaxanthin, violaxanthin, neoxanthin, and lutein (Fig. 3). The carotenoid compositions and contents differed significantly among the analyzed sweetpotato varieties. In SS8, eight carotenoids were detected except α-carotene and phytofluene (Fig. 3), among which β-carotene (approx. 36–53% of the total carotenoids) and β-cryptoxanthin (approx. 35–47%) were the dominant carotenoids. β-Branch carotenoid products accounted for 94.4–99.8% of the total carotenoids in SS8 (Additional file 5: Table S3, Additional file 1: Fig. S1). In XX, nine carotenoids excluding (E/Z)-phytoene, were detected, with α-carotene, γ-carotene, and phytofluene detected only at the first developmental stages (Fig. 3). β-cryptoxanthin (approx. 60–74%) was the dominant carotenoid, followed by zeaxanthin (approx. 16–23%), and thus β-branch carotenoid products accounted for 90.0–98.4% of the total carotenoids in XX (Additional file 5: Table S3, Additional file 1: Fig. S1). The content of β-cryptoxanthin showed a general increasing trend from 60 to 120 DAP, and a slight decrease at 135 DAP. The content of zeaxanthin generally continued to increase from 60 to 135 DAP (Additional file 5: Table S3). In XS18, seven carotenoids were detected, including α-carotene, β-carotene, β-cryptoxanthin, zeaxanthin, violaxanthin, neoxanthin, and lutein. β-cryptoxanthin (approx. 10.0–45.8%), zeaxanthin (approx. 0–35.4%), and violaxanthin (approx. 8.5–32.9%) comprised the predominant carotenoids (Additional file 1: Fig. S1), and β-branch products accounted for 75.0–97.8% of the total carotenoid content in XS18 (Additional file 5: Table S3). The contents of β-cryptoxanthin and zeaxanthin showed little variation during the first four stages but a decrease trend in the last two stages (Additional file 5: Table S3).

There were significant differences in the dynamic changes in carotenoid profiles during six tuberous root developmental stages of the sweetpotatoes with three flesh colors (Additional file 1: Fig. S1). In XS18, carotenoid accumulation could be divided into two stages. In the first stage (60–90 DAP), β-cryptoxanthin and zeaxanthin accounted for the higher proportion of carotenoids, peaking at 90 DAP (45.8% and 35.4%, respectively). In the second stage (105–135 DAP), the proportions of β-cryptoxanthin and zeaxanthin gradually decreased with the rapid increases in violaxanthin and neoxanthin. In XX, the proportion of β-cryptoxanthin increased from 60.1 to 73.7% with the development of tuberous roots. In SS8, β-carotene and β-cryptoxanthin accounted for the highest proportions of carotenoids, and the decrease of β-carotene was accompanied by an increase of β-cryptoxanthin during the development of tuberous roots (Additional file 1: Fig. S1).

### Differences in gene expression among the three sweetpotato cultivars

To explore the differences in expression levels of carotenoid pathway genes in the tuberous roots of the three cultivars*,* 10 carotenoid biosynthetic genes (*IbGGPS, IbPSY, IbPDS, IbZDS, IbCRTSIO, IbLCYB, IbLCYE, IbCHYB, IbCHYE,* and *IbZEP)* and three carotenoid catabolic genes (*IbCCD1, IbCCD4,* and *IbNCED3*) were analyzed using RT-qPCR (Fig. 1b).

In the tuberous roots of the three varieties, *IbGGPS, IbPSY, IbZDS, IbCHYB,* and *IbZEP* were highly expressed, compared to *IbLCYE* and *IbCHYE* which were expressed at low levels (Fig. 1b). *IbCHYB* and *IbZEP* are involved in the formation of β-branch carotenoids, while *IbLCYE* and *IbCHYE* are responsible for α-branch products. Therefore, such expression profiles of these biosynthesis genes are consistent with β-branch carotenoids being predominant in the three varieties (Fig. 3, Additional file 5: Table S3).

In SS8, the highest carotenoid content was correlated with the highest expression levels of *IbGGPS, IbPSY, IbCHYB,* and *IbZEP* at 60 DAP. In contrast, a lower carotenoid content was accompanied by lower expression levels of *IbGGPS* and *IbPSY* at 120 DAP (Fig. 1b). The expression of *IbCCD4,* a carotenoid catabolic gene, was lower in SS8 than that in XX and XS18 during the six tuberous root developmental stages. The expression of *IbNCED3* in SS8 was higher than that in XX (except at 75 DAP and 90 DAP) and XS18 (except at 60 DAP) during tuberous root development (Additional file 5: Table S4).

In XX, the total carotenoid content was lower than that in SS8. However, the expression levels of some carotenoid biosynthetic genes, such as *IbGGPS*, *IbZDS*, *IbLCYB*, *IbCHYB,* and *IbZEP*, were higher in XX than in SS8 at some developmental stages (Fig. 1b). For example, the expression levels of *IbZDS* at 90, 105, 120, and 135 DAP in XX were 1.6-, 4.5-, 2.0-, and 2.9-fold higher than those in SS8, respectively. The expression of *IbCCD4* in XX was higher than that in SS8 and XS18 (except at 75 DAP and 90 DAP) during tuberous root development (Additional file 5: Table S4).

In XS18, the expression levels of several carotenoid biosynthetic genes, including *IbGGPS, IbPSY, IbZDS,* and *IbCHYB,* were even higher than those in SS8 at some developmental stages, although the tuberous roots of XS18 contained only small amounts of carotenoids. For example, the expression levels of *IbPSY*, *IbZDS,* and *IbCHYB* at 120 DAP in XS18 were 2.9-, 1.8-, and 2.1-fold higher, respectively, than those in SS8. Additionally, we observed that the expression of *IbCCD4* in XS18 was higher than that in SS8 during all six tuberous root developmental stages (Additional file 5: Table S4).

### Correlation between the expression of carotenoid metabolic genes and carotenoid levels in tuberous roots of sweetpotato

**Fig. 4:**
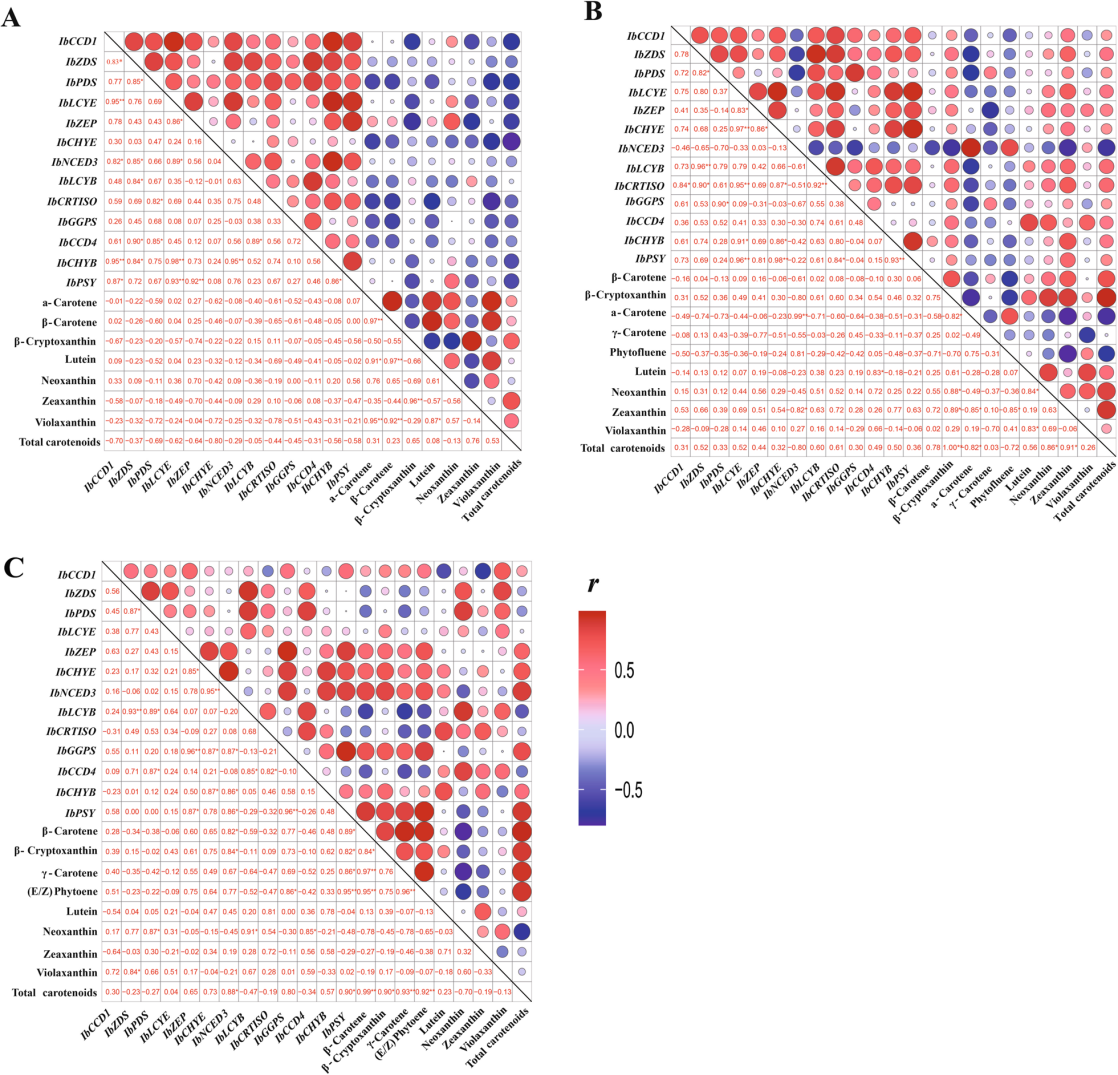
Heatmap of the correlations between thirteen carotenoid-related genes and specific carotenoids in the sweetpotato cultivars XS18 (**A**), XX (**B**), and SS8 (**C**). The *r* value represents the correlation coefficient, red indicates a positive correlation, blue indicates a negative correlation, and the circle size reflects credibility. A larger circle indicates a smaller *p* value and stronger correlation. * represents *p* < 0.05, and ** represents *p* < 0.01

Pearson correlation analysis was performed to evaluate the relationship between the expression of carotenoid metabolic genes and carotenoid contents. Pearson’s *p* values were visualized as a correlation matrix (Fig. 4). As shown in Fig. 4a, no significant correlation was observed between the expression levels of 13 carotenoid metabolic genes and contents of the specific carotenoids detected in XS18 (*p* < 0.05, *r* < 0.5).

In XX, there was a higher positive correlation between the β-cryptoxanthin content and the expression of *IbLCYB* (*r* = 0.61). Moreover, the content of zeaxanthin was positively correlated with the expression of *IbPSY*, *IbZDS*, *IbLCYB*, and *IbCHYB* (*r* ranged from 0.63 to 0.77), respectively (Fig. 4b). The total carotenoid content was positively correlated with the expression of *IbZDS*, *IbLCYB*, and *IbCHYB* (*r* ranged from 0.50 to 0.60) (Fig. 4b).

In SS8, the expression of *IbPSY* was positively correlated with the contents of β-carotene, β-cryptoxanthin, γ-carotene, (E/Z)-phytoene, and total carotenoids (*p* < 0.05, *r* ranged from 0.82 to 0.95), respectively (Fig. 4c). The contents of both β-cryptoxanthin and the total carotenoids were positively correlated with the expression of *IbGGPS* and *IbCHYB* (*r* ranged from 0.57 to 0.80) (Fig. 4c). The expression of *IbNCED3* had strong positive correlations with the contents of β-carotene, β-cryptoxanthin, and the total carotenoids (*p* < 0.05,* r* ranged from 0.82 to 0.88) (Fig. 4c).

### Isolation and analysis of *IbCCD4*

**Fig. 5:**
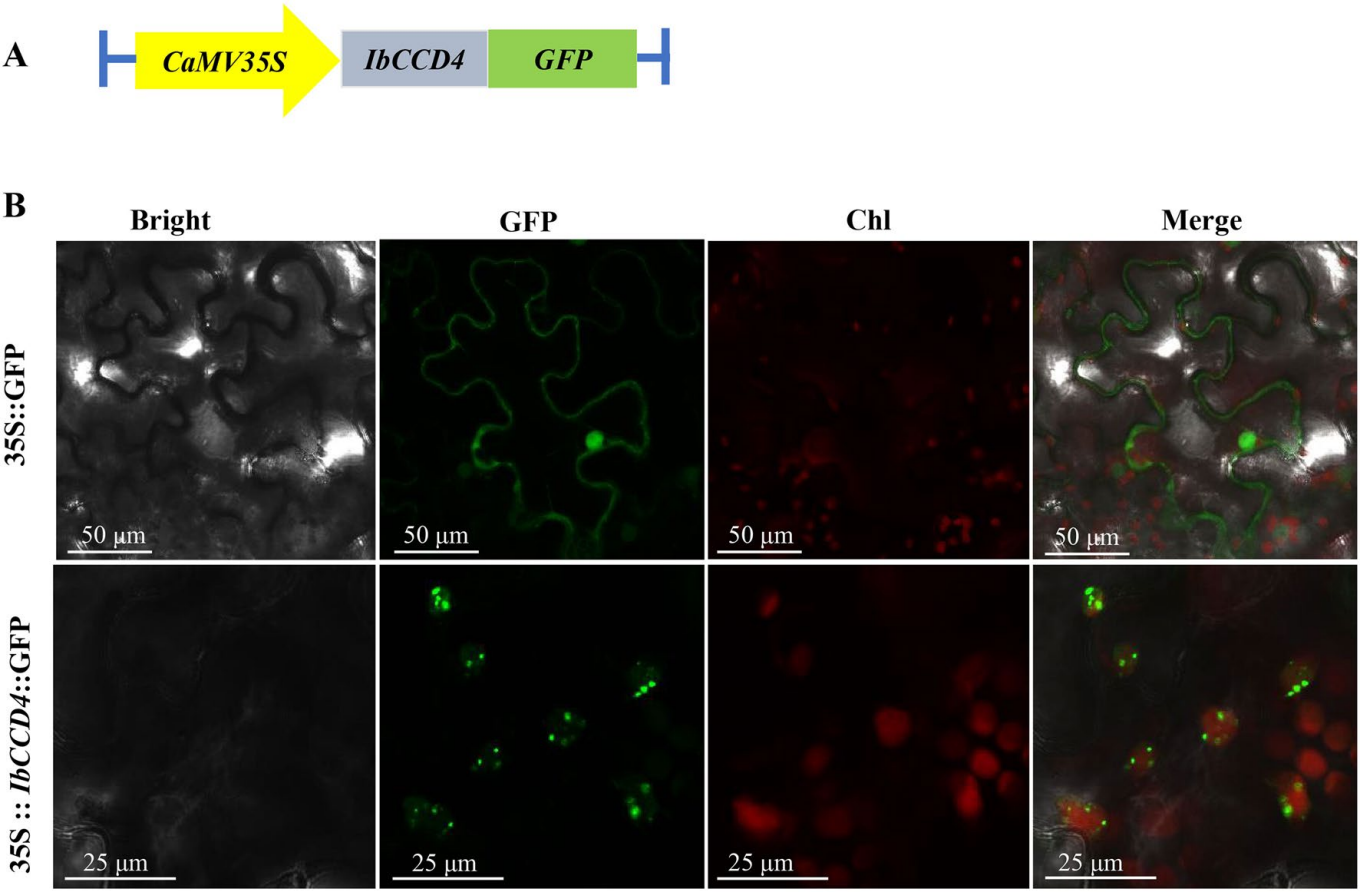
Subcellular localization of IbCCD4. **A** Diagram of the IbCCD4-GFP construct. **B** Subcellular localization of the 35S::IbCCD4::GFP fusion protein. Chl, chloroplast

IbCCD4 may play an important role in carotenoid accumulation in sweetpotato tuberous roots. Here, we cloned the *IbCCD4* gene and characterized its encoded protein. A 1785 bp of open reading frame sequence of *IbCCD4* (GenBank accession number OM674440) was isolated from the cDNA of XS18 tuberous roots. The amino acid sequence of IbCCD4 contains the four conserved histidine, and glutamate or aspartate residues (Additional file 2: Fig. S2). Phylogenetic analysis (Additional file 3: Fig. S3) showed that the IbCCD4 protein was most closely related to a CCD4 of *Ipomoea triloba,* an ancestor of sweetpotato. The online prediction of subcellular localization suggested that IbCCD4 is localized in the chloroplast. A 35S::*IbCCD4*::GFP vector was constructed (Fig. 5a) and transformed into tobacco. As shown in Fig. 5b, the green fluorescence signal overlapped with the red autofluorescence of chlorophyll, suggesting the chloroplast localization of IbCCD4.

### The *IbCCD4*-overexpressing tuberous roots showed a decreased total carotenoid content

**Fig. 6:**
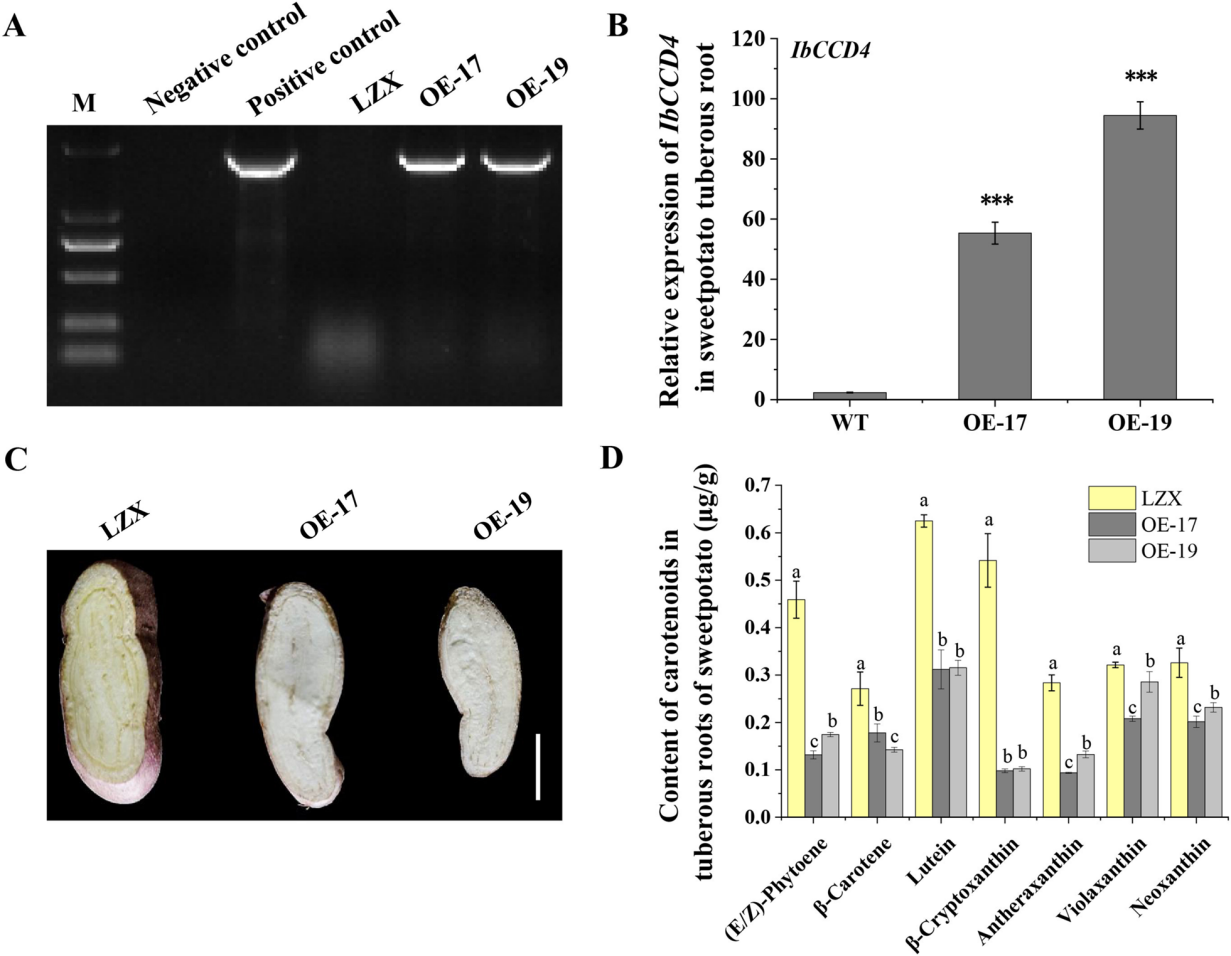
Overexpression of *IbCCD4* in sweetpotato reduces the accumulation of carotenoids in the tuberous roots. **A** Polymerase chain reaction amplification of *IbCCD4* from the cDNA of non-transgenic plants (LZX) and transgenic sweetpotato lines (OE-17 and OE-19). M, marker; Negative control, H_2_O; Positive control, the 35S::IbCCD4::GFP vector. **B** Relative expression levels of *IbCCD4* in transgenic sweetpotato lines (OE-17 and OE-19). Data are means ± standard deviation (SD) (*n* = 3). A significant difference was performed between WT and OE. ***p < 0.001. **C** Phenotype of non-transgenic sweetpotato (LZX) and transgenic sweetpotato lines (OE-17 and OE-19) at 90 DAP. Scale bars = 2 cm. **D** Contents of carotenoids in the tuberous root of non-transgenic (LZX) and transgenic sweetpotato lines (OE-17 and OE-19). Data are means ± standard deviation (SD) (*n* = 3). Different letters represent significant differences. *p* < 0.05

To ascertain the direct role of *IbCCD4* on the carotenoid accumulation in sweetpotato, the 35S::*IbCCD4*::GFP vector was genetically transformed into an sweetpotato embryogenic callus suspension derived from cv, Lizixiang (LZX, yellow fleshed) to obtain the transgenic sweetpotato lines. The transgenic status of *IbCCD4*-overexpressing lines (OE-17 and OE-19) was confirmed by PCR (Fig. 6a), and overexpression of *IbCCD4* was detected using RT-qPCR (Fig. 6b). The transcript levels of *IbCCD4* in OE-17 and OE-19 were 60- and 90-fold higher, respectively, than that in non-transgenic sweetpotato (LZX). In contrast to the yellow flesh color of LZX, the flesh color of the two transgenic lines was white (Fig. 6c). Corresponding to the color change, the contents of the total carotenoids in the OE-17 and OE-19 decreased by 56.8% and 51.1%, respectively, compared to LZX (Fig. 6d; Additional file 5: Table S5). Moreover, the contents of seven carotenoids [(E/Z)-phytoene, β-carotene, lutein, β-cryptoxanthin, antheraxanthin, violaxanthin, and neoxanthin] were significantly decreased in the tuberous roots of transgenic lines OE-17 and OE-19 compared to those in LZX. These transgenic analysis indicated that IbCCD4 is a key player in the determination of carotenoid accumulation in sweetpotato tuberous roots.

### Overexpression of *IbCCD4* caused changes in the expression of several endogenous carotenogenic genes

**Fig. 7:**
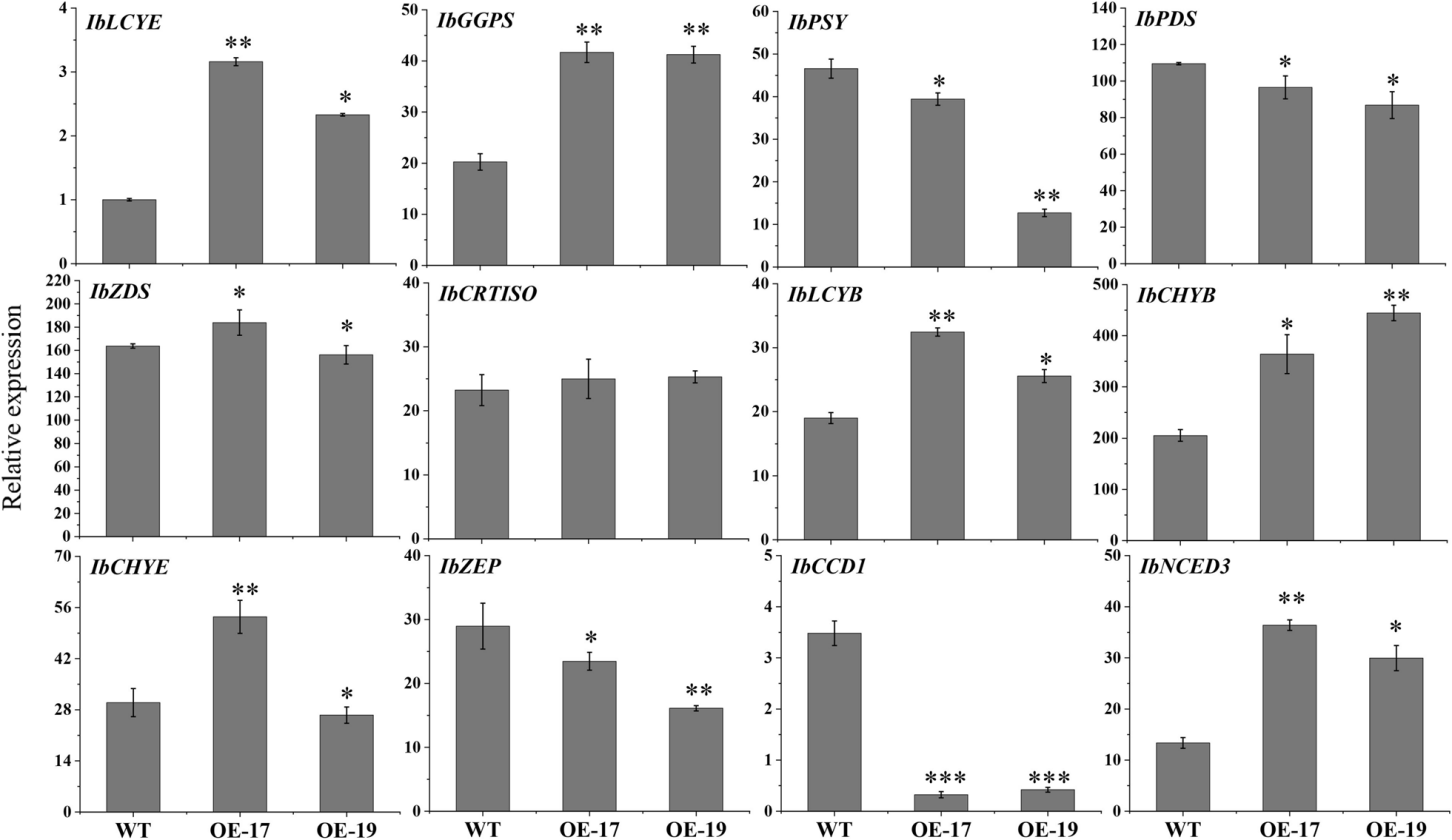
Relative expression levels of carotenoid-related genes in tuberous roots of *IbCCD4-*overexpressing lines by RT-qPCR. *IbActin* was used as an internal control. The expression of *IbLCYE* in LZX was used for calibration. Data are means ± standard deviation (SD) (*n* = 3). A significant difference was observed between WT and OE. ***p < 0.001; **p < 0.01; *p < 0.05

To reveal the possible impact of *IbCCD4* overexpression on other endogenous genes, the transcript levels of genes involved in the carotenoid metabolic pathway were analyzed in control and *IbCCD4*-overexpressing sweetpotato (OE-17 and OE-19) (Fig. 7).

Compared to the control (WT), the expression levels of *IbPSY*, *IbPDS*, *IbZEP,* and *IbCCD1,* but not *IbCRTISO,* were decreased significantly in OE-17 and OE-19. The expression levels of *IbGGPS*, *IbLCYE*, *IbLCYB*, *IbCHYB,* and *IbNCED3* were significantly increased in OE-17 and OE-19 compared to WT. Overall, overexpression of *IbCCD4* led to a significant change in expression levels of key endogenous genes related to the carotenoid pathway.

### The transcription factors IbWRKY20 and IbCBF2 interacted with the *IbCCD4* promoter

**Fig. 8:**
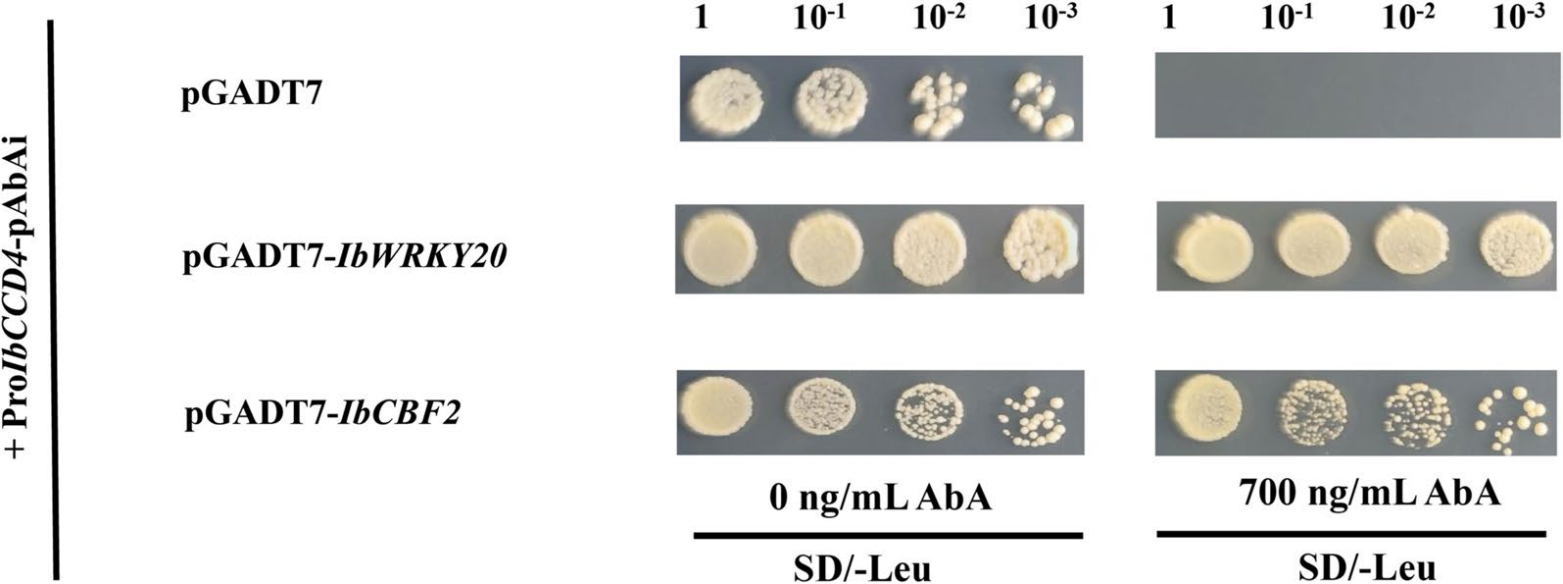
Y1H assay showing the physical interaction of the *IbCCD4* promoter with IbWRKY20 and IbCBF2

To investigate potential transcription factors that directly regulate *IbCCD4,* we isolated the *IbCCD4* promoter and used it in a yeast one-hybrid assays (Y1H) library screening. The 1932-bp *IbCCD4* promoter sequence (GenBank accession number ON920927) harbors a series of *cis*-acting regulatory elements associated with the stress responses, including the ABA response element (ABRE), ACGT element, W-box, T/G-box, as well as CGTCA and TGACG motifs (Additional file 5: Table S6, Additional file 4: File S2). We were intrigued by the ABRE element as various transcription factors recognize this element in ABA signaling. We thus screened a sweetpotato cDNA library using a 260-bp *IbCCD4* promoter fragment containing an ABRE element, as well as a W-box, T/G-box, and ACGT element, as the bait (Additional file 4: File S2). A total of 40 clones were obtained from the cDNA library screening. DNA sequencing of the 40 clones identified two transcription factor genes, XP_031092613.1 and XP_031098635.1. XP_031092613.1, was annotated as a WRKY transcription factor. XP_031098635.1 is homologous to the *Arabidopsis* gene (AT4g25470), belonging to the drought response element binding factor (DREB1s/CBF) family. The two genes were named as *IbWRKY20* (GenBank accession number ON920924) and *IbCBF2* (GenBank accession number ON920925). To further confirm the interaction of the two transcription factors with the *IbCCD4* promotor, the vectors pGADT7, pGADT7-*IbWRKY20,* and pGADT7-*IbCBF2* were separately transformed into a Y1H strain integrated with the promoter sequence of *IbCCD4* (Pro*IbCCD4*-pAbAi), and the yeast was then plated on synthetic dextrose (SD)/-Leu medium supplemented with or without 700 ng/mL AbA. The results showed that the pGADT7-*IbWRKY20* and pGADT7-*IbCBF2* transformants grew well on a medium containing 700 ng/mL AbA, whereas the negative control yeast (containing the pGADT7 vector) did not (Fig. 8). These results indicate that the proteins IbWRKY20 and IbCBF2 activate the *IbCCD4* promoter.

## Discussion

### Three varieties of sweetpotato with different flesh colors represent different carotenoid profiles

As high-value and health-promoting natural products, carotenoid contents and their compositions were well characterized in the tuberous roots of sweetpotato [28, 31–34]. However, there is a lack of data describing the dynamic changes in carotenoid profiles during different tuberous root developmental stages of sweetpotato varieties with different flesh colors. We found that the compositions, contents, and proportions of carotenoids differed among the three sweetpotato varieties during tuberous root development (Fig. 3, Additional file 1: Fig. S1). These differences imply that carotenoid metabolism in sweetpotato tuberous roots may be based on the genetics of the varieties, as well as the regulatory mechanisms of tuberous root development and external environmental stimuli, similar to what have been reported in other plant species [35–37].

The contents of dry matter and starch increase during sweetpotato tuberous root development. The harvest time of sweetpotato tuberous roots is generally at 120 DAP, when the tuberous root dry matter and starch contents are the highest [38–40]. In our study, β-carotene (approx. 36.3–53.4% of the total carotenoids) and β-cryptoxanthin (approx. 35.0–47.4%) were found to be the dominant carotenoids in SS8 (Fig. 3). There is a positive correlation between the contents of β-carotene and β-cryptoxanthin (Fig. 4c). The content of β-carotene decreased during tuberous root development except at 120 DAP (Additional file 5: Table S3), which was consistent with the change in the total carotenoid content. These results indicate that the dynamic changes in the total carotenoid content of SS8 are due to the changes in β-carotene contents. The content of β-carotene decreased gradually during tuberous root development in SS8, which was largely in agreement with previous studies, showing that storage root dry matter and starch content are negatively correlated with β-carotene content in the tuberous roots of sweetpotato [41–43]. Our data showed that the total carotenoid contents were higher at 120 DAP for all three sweetpotato varieties. This is closely associated with abundant carbon sources (e.g., isoprenoid precursors) and the prolonged accumulation time at harvest.

We observed that β-branch carotenoid products were dominant in SS8, accounting for 94.4–99.8% of the total carotenoids (Additional file 5: Table S3), similar to what has been described by Amoanimaa-Dede et al. [28]. Moreover, β-branch carotenoid products were also the predominant components of XX and XS18, accounting for 91.8–99.0% and 75.0–97.8% of the total carotenoids, respectively (Additional file 5: Table S3). β-carotene (approx. 36.3–53.4%) and β-cryptoxanthin (approx. 35.0–47.5%) were the main carotenoids in SS8, and β-cryptoxanthin (approx. 60.1–73.7%) and zeaxanthin (approx. 15.7–22.6%) were dominant in XX (Fig. 3). This is inconsistent with a report by Ishiguro et al. [33], who used high-performance liquid chromatography (HPLC) to show that β-carotene 5,8;5′,8′-diepoxide (approx. 32–51%) and β-cryptoxanthin 5′,8′-epoxide (approx. 11–30%) are the dominant carotenoids in the tuberous roots of eight yellow-fleshed sweetpotato cultivars and that β-carotene (approx. 80–92%) is dominant in the tuberous roots of four orange-fleshed sweetpotato cultivars. UPLC-APCI-MS/MS analysis has shown that β-cryptoxanthin and β-carotene are the predominant carotenoids in yellow-fleshed sweetpotato “Baishu” and white-fleshed sweetpotato “shangshu19,” respectively [32]. UPLC-APCI-MS/MS analysis has also determined that β-cryptoxanthin and lutein are enriched in yellow-fleshed sweetpotato “Jieshu 95–16” and white-fleshed sweetpotato “shangshu19,” respectively [31]. These inconsistent results from different studies could be related to variations in genetic inheritance, growing environments [36], and analytical methods.

### Carotenoid catabolic enzymes significantly affect carotenoid accumulation in the three varieties

Many studies have shown the important roles of carotenoid biosynthetic genes, such as *GGPS, PSY, LCYB, ZDS,* and *CHYB* in carotenoid accumulation. Overexpression of *IbGGPS* improves the total carotenoid content in *Arabidopsis thaliana* leaves [11]. The expression level of *IbPSY* is higher in yellow-fleshed sweetpotato than in the white-fleshed variety [32]. The β-carotene content is associated with the expression of *IbPSY* in sweetpotato [44]. The up-regulation of *PSY*, *LCYB,* and *CHYB* leads to a high accumulation of the total carotenoid content in red-fleshed loquat [45]. *CHYB* (g1548 and g953) in the yellow-fleshed sweetpotato expresses higher than that in the white-fleshed variety [32]. Overexpression of *IbZDS* increases the contents of β-carotene and lutein in sweetpotato [29]. However, carotenoid catabolic enzymes also function in determining carotenoid content in plants. The carotenoid catabolic enzyme CCD4 is involved in the degradation of carotenoids in flowers, fruits, and tubers of various plants [18–21]. However, whether IbCCD4 affects carotenoid composition and flesh color of tuberous roots remained unclear.

In SS8, the total carotenoid content was the highest at 60 DAP (Fig. 2b), which was consistent with the higher expression levels of *IbGGPS, IbPSY,* and *IbCHYB*. And the expression of these carotenoid biosynthesis genes was 5.4-, 11.6-, and 7.2-fold higher in SS8 than in white-fleshed XS18, respectively (Fig. 1b, Additional file 5: Table S4). The contents of β-cryptoxanthin and total carotenoids in SS8 were positively correlated with the expression of *IbGGPS* and *IbCHYB*, and the expression of *IbPSY* was positively correlated with the contents of β-carotene and β-cryptoxanthin, respectively (Fig. 4c). These results suggest that the contents of dominant β-carotene and β-cryptoxanthin in SS8 are related to the higher expression levels of the carotenoid biosynthesis genes *IbGGPS, IbCHYB*, and especially *IbPSY.* The expression of *IbCCD4* in SS8 was lower than that in XX and XS18. Such weak expression of *IbCCD4* might lead to less degradation of carotenoids and therefore may contribute to the higher content of total carotenoids in SS8 compared to XX and XS18.

In XX, β-cryptoxanthin content was positively correlated to the *IbLCYB* expression (*r* = 0.61), while the content of zeaxanthin was positively correlated with the expression of *IbPSY*, *IbZDS*, *IbLCYB*, and *IbCHYB* (Fig. 4b). Moreover, the total carotenoid content was positively correlated with the expression of *IbZDS*, *IbLCYB*, and *IbCHYB* (Fig. 4b). These results indicate that the dominant accumulation of β-cryptoxanthin and zeaxanthin in XX is the result of higher transcript levels of carotenoid biosynthesis genes (e.g., *IbPSY*, *IbZDS*, *IbLCYB*, and *IbCHYB*) during tuberous root development. By comparison, the expression of *IbCCD4* in XX was higher than that in SS8, possibly contributing to the lower content of total carotenoids in XX than in SS8.

In the low-carotenoid XS18 and the medium-carotenoid XX, *IbGGPS, IbPSY, IbZDS,* and *IbCHYB* during the developmental stages were consistently expressed at the same or even higher level than that in the high-carotenoid SS8 (Fig. 1b). These results were consistent with a previous comparison in white-fleshed carrots [46]. The expression of *IbCCD4* in XS18 and XX was higher than that in SS8, suggesting that *IbCCD4* plays an important role in the low carotenoid content in the flesh of tuberous roots.

*IbNCED3* encodes an NCED enzyme that cleaves 9-*cis*-epoxycarotenoid (violaxanthin and neoxanthin) to produce the phytohormone abscisic acid (ABA) [17]. *IbNCED3* was expressed at a relatively high level in the three varieties (Fig. 3b), which may result in the low accumulation of carotenoids in XX and XS18. Similar results have been observed previously [7, 32, 47]. For example, the low total carotenoid content in the yellow watermelon (*Citrullus lanatus*) was shown to be caused by the high expression levels of *ClNCEDs* [7]*.* The expression levels of *NCED3* and *NCED4* were higher in the white-fleshed sweetpotato than in the yellow-fleshed variety [32]. Suppression of *SlNCED1* increased the contents of lycopene and β-carotene in tomato (*Solanum lycopersicum*) [47]. In SS8, *IbNCED3* was consistently expressed at a high level (Fig. 1b) during tuberous root development. In SS8, β-carotene and β-cryptoxanthin were the dominant carotenoids, but there was only a small amount of violaxanthin (0.5–1.1%) and neoxanthin (0.1–0.4%) (Additional file 5: Table S3, Additional file 1: Fig. S1). The high expression of *IbNCED3* may not significantly affect the high carotenoid content of SS8. Further experiments are needed to conclusively confirm whether the down-regulation of *IbNCED3* can enhance the total carotenoid content in the tuberous roots of sweetpotato.

It is known that the accumulation of carotenoids in plants is mainly determined by both biosyntheses (regulated by biosynthesis genes, e.g., *CHYB*, *ZDS,* and *LCYE*) and catabolism (regulated by CCDs, e.g., *NCED3* and *CCD4*). We found that in white-fleshed XS18, both the biosyntheses and catabolism of carotenoids are active, evidenced by the fact that the *IbCCD4* expression was positively correlated with those of *IbZDS*, *IbLCYE,* and *IbCHYB*, whereas the expression of *IbNCED3* was positively correlated with those of *IbZDS*, *IbLCYE,* and *IbCHYB* (Fig. 4a). In orange-fleshed (high-carotenoid) SS8, the carotenoid biosynthetic genes were highly expressed, while the carotenoid degrading gene *IbCCD4* was expressed at a significantly lower level compared to the yellow-fleshed (medium-carotenoid) XX and the white-fleshed (low-carotenoid) XS18. In addition, in yellow-fleshed XX, the expression of *IbCCD4* and *IbNCED3* was not correlated with that of *IbZDS*, *IbLCYE,* and *IbCHYB*, although the total carotenoid content was positively correlated with the expression of *IbZDS*, *IbLCYB*, and *IbCHYB* (Fig. 4b). In orange-fleshed SS8, the expression of *IbCCD4* and *IbNCED3* was not correlated with those of *IbZDS*, *IbLCYE,* and *IbCHYB*, and the expression of *IbCHYB* was positively correlated with the total carotenoid content. There was no correlation between the total carotenoid content and the expression of *IbZDS* and *IbLCYB* (Fig. 4c). Our results indicate that both biosynthetic and metabolic genes are expressed in all three varieties. However, the amplitudes of expression vary significantly among them. The underlying mechanisms for balance between biosyntheses and catabolism of carotenoids may involve both gene regulation and carotenoid metabolism, and also may be genotype-specific because the sweetpotato genome exhibits hexaploidy and high heterozygosity [48].

Overall, our results suggest that the high-level accumulation of carotenoids in SS8 is due to the high expression of the carotenoid biosynthetic genes *IbGGPS, IbPSY,* and *IbCHYB*, especially *IbPSY*, and the low expression of the carotenoid metabolic gene *IbCCD4*. In XX and XS18, the higher expression of *IbCCD4* may contribute to the lower content of total carotenoids than that in SS8.

### *IbCCD4* overexpression reduces carotenoid contents in sweetpotato tuberous roots

The flesh color of tuberous roots in *IbCCD4-*overexpressing sweetpotato lines OE-17 and OE-19 became white due to the decreased content of total carotenoids (Fig. 6c). The total carotenoids and seven specific carotenoids [(E/Z)-phytoene, β-carotene, lutein, β-cryptoxanthin, antheraxanthin, violaxanthin, and neoxanthin] significantly decreased in the *IbCCD4-*overexpressing transgenic tuberous roots (Fig. 6d). These results are in good agreement with the gene expression profiling of the three varieties.

CCD4s from different species display varied carotenoid substrate specificity. For example, the main substrate of CsCCD4 from *Camellia sinensis* is β-carotene [49]. In *citrus*, CitCCD4 cleaves β-cryptoxanthin and zeaxanthin to form β-citraurin [50], and CitCCD4b cleaves β-carotene to produce β-apo-8′-carotenal [51]. DcCCD4 degrades α- and β-carotene to produce α- and β-ionone in *Daucus carota* [52]. Park et al. [22] cloned an *IbCCD4* gene (KM973214) from sweetpotato, which shows 96.8% identity to the *IbCCD4* in the present study. They showed that β-carotene is the main substrate of IbCCD4 in vitro. In the present study, the increased expression of *IbCCD4* in OE-17 and OE-19 is associated with a significant decrease of seven carotenoids [(E/Z)-phytoene, lutein, β-carotene, β-cryptoxanthin, antheraxanthin, violaxanthin, and neoxanthin] (Fig. 6). Whether these carotenoids are substrates of IbCCD4 needs to be verified in future studies.

Another reason for the decreased content of carotenoids in *IbCCD4*-overexpressing tuberous roots may be due to changes in the expression of endogenous carotenogenic genes. The expression of *PSY* constitutes a bottleneck in the carotenoid metabolic pathway [13, 15, 36]. In the present study, the down-regulation of *IbPSY* in *IbCCD4-*overexpressing tuberous roots (Fig. 7) may be responsible for the lower content of the upstream precursor phytoene, resulting in a decreased downstream carotenoid content. Moreover, the up-regulation of *IbNCED3* in *IbCCD4-*overexpressing tuberous roots (Fig. 7) may also contribute to a lower total carotenoid content, as previously reported [7, 47].

Carotenoid contents were determined based on the transcript levels of not only carotenoid biosynthetic genes but also catabolic genes. When the expression of *IbCCD4* was increased, the degradation of carotenoids was accelerated, resulting in a decrease in the total carotenoid content, even though the expression of some carotenoid biosynthetic genes was increased (Fig. 7). The overexpression of *IbCCD4* likely affected the metabolic flux of carotenoids which, in turn, altered the expression of corresponding genes through feedback regulation.

### IbCCD4 may be involved in abiotic stress responses

CCD4 is a key regulator of carotenoid turnover in different organs of various plant species [49–52]. In addition, the catabolic products of CCD4 are important bioactives involved in abiotic stress responses [53, 54]. It is thus reasonable that *IbCCD4* is regulated by transcription factors involved in stress signal transduction. In an attempt to identify regulators of *IbCCD4*, a Y1H screening showed that IbWRKY20 and IbCBF2 activated the *IbCCD4* promoter (Fig. 8). The outcome is supported by the presence of WRKY-binding motif (W-box) and CBF-binding motif (A/GCCGAC) in the *IbCCD4* promoter. The WRKYs comprise a large family of transcription factors in higher plants, playing important roles in disease resistance and response to abiotic stresses [55–57]. WRKYs are key components in ABA signaling. *CBF* genes encode transcription factors that play important roles in plant stress responses. The *Arabidopsis AtCBF1, AtCBF2,* and *AtCBF3* are involved in cold, salt, and drought stress responses [58–60]. *CBF* expression responses to the induction of the stress phytohormone ABA. We have found that the overexpression of *IbCCD4* decreases salt resistance in *Arabidopsis thaliana* [61]. The discovery of the direct activation of the *IbCCD4* promoter by IbWRKY20 and IbCBF2 provided evidence, perhaps for the first time, for ABA regulation of abiotic stress through modulating carotenoid metabolism. Characterization of these two transcription factors will expand our understanding of how environmental cues affecting carotenoid accumulation.

## Conclusion

The present study reveals that both biosynthetic and catabolic genes work in concert to balance carotenoid accumulation in sweetpotato tuberous roots. IbCCD4 is identified as a key player in the determination of total carotenoid contents in sweetpotato. Moreover, activation of the *IbCCD4* promoter by IbWRKY20 and IbCBF2 indicated a possible regulation mechanism for carotenoid accumulation by ABA signaling. These findings shed light on the molecular mechanisms underlying carotenoid accumulation in tuberous roots and insinuated that *IbCCD4* can be used as a target for genetic engineering to breed new sweetpotato variety enriched with carotenoids to meet market demand for natural carotenoids.

## Materials and methods

### Plant materials and sampling

Three sweetpotato varieties (Fig. 2a), including the white-fleshed sweetpotato cv. “Xushu18” (WFSP cv. XS18), the yellow-fleshed sweetpotato cv. “Xinxiang” (YFSP cv. XX), and the orange-fleshed sweetpotato cv. “Sushu8” (OFSP cv. SS8), were obtained from the Sweetpotato Research Institute of the China Agriculture Academy of Science (Xuzhou, Jiangsu, China). The cutting vines (approximately 10 cm) were grown at Shanxi Agricultural University (Taigu, China) under normal conditions in May 2019. Five tuberous roots from five individual plants of each cultivar were mixed as one biological replicate, with sampling conducted at 60, 75, 90, 105, 120, and 135 days after planting (DAP). There were three replicates of each variety at each time point. The samples were frozen quickly in liquid nitrogen after washing, peeling, and cutting into small pieces and stored at − 80 °C.

### Determination of total carotenoid contents in tuberous roots of sweetpotato

The carotenoid content was determined by the reported method with some modifications [62]. Frozen samples were ground into powder. A total of 500 mg of powder was weighed and extracted using 20 mL of acetone for 3 h at 4 °C in the dark until the tissue was deprived of color. After centrifugation at 4000 rpm/min for 10 min, the absorbance of the supernatant was measured at 454 nm by spectrophotometry. The carotenoid content was calculated using the formula A_454_/m, where A_454_ is the absorbance at 454 nm and m indicates the sample weight.

### Metabolomic analysis

The extraction, identification, and quantitative analysis of carotenoid metabolites in 54 samples (from three varieties at six developmental stages, and three replicates) were performed by Wuhan MetWare Biotechnology Co., Ltd. (www.metware.cn). Briefly, the freeze-dried tuberous root samples were ground and extracted. Sample extracts were filtered and analyzed by UHPLC-APCI-MS/MS. All carotenoid metabolites were annotated in the MetWare database and quantified using multiple reaction monitoring (MRM). Metabolite data analysis was performed with Analyst 1.6.3 software (AB Sciex). The detailed methodology [63] is provided in Additional file 4: File S1.

### Gene expression analysis by RT-qPCR

Total RNA was extracted from frozen tuberous roots sampled at 60, 75, 90, 105, 120, and 135 DAP using an RNA extraction kit (TAKARA Biotechnology, Dalian, Liaoning, China). The RNA integrity was evaluated by 1% agarose gel electrophoresis. The RNA concentrations and quality were measured with a NanoDrop 2000C system (Thermo Scientific, Waltham, Massachusetts, USA). First-strand cDNA was synthesized from 1 μg of total RNA according to the instructions of the PrimeScript™ RT reagent kit with gDNA Eraser (TAKARA Biotechnology, Dalian, Liaoning, China). The reaction was performed in a volume of 20 μL, at 42 °C for 15 min and 85 °C for 5 s. All RT-qPCR experiments were performed on a CFX96 PCR system (Bio-Rad, USA) using SYBR^®^ Premix Ex Taq™ (TAKARA Biotechnology, Dalian, Liaoning, China), with a reaction volume of 10 μL, including 1 μL cDNA, 5 μL 2 × SYBR^®^ Premix Ex Taq™, 3.6 μL ultrapure water, and 0.2 μL each primer. The reaction conditions were as follows: an initial denaturation step of 95 °C for 3 min, followed by 40 cycles of amplification (95 °C/10 s, 60 °C/30 s, and 72 °C/20 s). The melting curve was obtained by heating the amplicon from 60 to 95 °C. Carotenoid biosynthetic genes (*IbGGPS, IbPSY, IbPDS, IbZDS, IbCRTSIO, IbLCYB, IbLCYE, IbCHYB, IbCHYE,* and *IbZEP*) and carotenoid catabolic genes (*IbCCD1, IbCCD4,* and *IbNCED3*) were amplified with gene-specific primers designed using Primer Premier 5.0. The primer sequences are listed in Additional file 5: Table S1. The *IbActin* gene (AY905538) was used as an internal reference standard [11, 64]. The relative expression levels of the genes were calculated using the 2^−ΔΔCT^ method [65]. The expression level of *IbCCD1* at 60 DAP in WFSP cv. XS18 was used as the calibration standard for constructing the heatmap using TBtools software [66].

### Analysis of correlation coefficients

Pearson correlation coefficients were calculated with the R package ggcor to evaluate correlations between the expression of thirteen genes involved in the carotenoid metabolic pathway and carotenoid contents [67]. Statistical significance was tested by one-way analysis of variance (ANOVA) with the least significant difference (LSD) method in R language (version 3.6.1).

### Gene cloning and sequence analysis of *IbCCD4*

Using the cDNA of the XS18 tuberous root as the template, PCR amplification was performed with specific primers (Supporting Information, Table S1). After an initial denaturation step at 95 °C for 4 min, 35 cycles were performed each with 30 s of denaturation at 95 °C, followed by 30 s of annealing at 60 °C and 60 s of extension at 72 °C. The PCR product was purified and cloned into the pMD19-T vector (TaKaRa). The open reading frame (ORF) sequence of *IbCCD4* was obtained by sequencing. The amino acid sequence of IbCCD4 (OM674440) was aligned with CCD4 sequences from nine other plant species obtained from the NCBI database (https://www.ncbi.nlm.nih.gov/) using ClustalW software. Phylogenetic analysis was performed using MEGA7 via the neighbor-joining (NJ) method, with 1000 replicates [68].

### Subcellular localization of the IbCCD4 protein

The subcellular localization of the IbCCD4 protein was predicted with the online tool Plant-mPLoc (http://www.csbio.sjtu.edu.cn/bioinf/plant-multi/). The ORF sequence of *IbCCD4* lacking the stop codon was PCR amplified with specific primers (Additional file 5: Table S1) harboring *Kpn*I and *Xba*I restriction sites and then inserted into the pCambia1300 vector, which contains the *GFP* and hygromycin phosphotransferase II (*hptII*) genes. The constructed vector, 35S::*IbCCD4*::*GFP*, was transformed into *Agrobacterium tumefaciens* strain GV3101, and subcellular localization analysis was performed in tobacco leaves following a previously reported method [69]. The injected tobacco plants were placed in a light incubator in the dark for 24 h, followed by a 16 h light and 8 h dark period for a total of 48 h (26 °C, 9000 lx). GFP fluorescence was observed using a Leica TCS SP8 confocal microscope.

### Generation of *IbCCD4*-overexpressing transgenic sweetpotato

Lizixiang (LZX, yellow-fleshed sweetpotato) is one of the finest varieties which have been established an efficient transformation system [70]. Following the reported method [70], the 35S::*IbCCD4*::*GFP* plasmid was transformed into an embryogenic suspension callus induced from LZX by *Agrobacterium tumefaciens*-mediated transformation (strain EHA105). The transformed callus was plated on an MS medium containing 10 mg/L hygromycin until plantlets were regenerated. Genomic DNA from regenerated plants was extracted from leaves and used for PCR detection with specific primers (Additional file 5: Table S1). The relative expression levels of *IbCCD4* and carotenoid metabolic genes in non-transgenic plants and LZX were analyzed by RT-qPCR.

### Quantification of carotenoids in the tuberous roots of transgenic sweetpotato

Tuberous root samples of LZX and transgenic sweetpotato were collected at 90 DAP. Samples from three tuberous roots of each transgenic line were mixed as one biological replicate. There were three replicates for each transgenic line. Carotenoid composition and content were determined using UHPLC-APCI-MS/MS by MetWare Biological Science and Technology Co., Ltd., (Wuhan, China) according to protocols reported by Zhou et al. [63]. The total carotenoid content recorded for each sample was the sum of the specific carotenoids detected.

### Yeast one-hybrid analysis of proteins that activate the *IbCCD4* promoter

The promoter sequence of *IbCCD4* (2.0 kb upstream of the transcription start site) was cloned by PCR using specific primers (Additional file 5: Table S1) by PCR and then sequenced. The potential *cis*-acting element of the *IbCCD4* promoter was analyzed in the PlantCARE database (http://bioinformatics.psb.ugent.be/webtools/plantcare/html/). A Y1H was conducted using the Matchmaker Gold Yeast One-Hybrid Library Screening System (Clontech, Mountain View, CA, USA). An artificial synthetic sequence of 260-bp sequence of the *IbCCD4* promoter was ligated into the pAbAi vector to construct the bait vector (Pro*IbCCD4*-pAbAi). The bait vector was transformed into the Y1H golden strain and screened using the minimal inhibitory concentration of aureobasidin A (AbA). The cDNAs of sweetpotato tuberous roots were cloned into the pGADT7 vector to construct a cDNA yeast library (OE Biotech Company, Qingdao, China), which was used to screen for proteins that interacted with the bait fragment of the *IbCCD4* promoter. Thereafter, the screened interacting proteins were sequenced with the T7 primer, and sequence alignment was conducted in the NCBI database (https://www.ncbi.nlm.nih.gov/) for functional annotation. The proteins were named according to sequence similarity with homologous Arabidopsis genes (https://www.arabidopsis.org/index.jsp). Protein‒DNA interactions were confirmed on synthetic dextrose (SD)/-Leu medium supplemented with 700 ng/mL AbA. The empty vector pGADT7 was used as a negative control.

### Statistical analysis of data

All data were analyzed with SPSS software (Chicago, IL, United States, version 8.0) by using Student’s *t* test or one-way ANOVA and the least significant difference (LSD) test. All figures were generated with Origin software (Northampton, MA, USA, version 2019).

## Supplementary Information

**Additional file 1. Fig. S1**: Dynamic changes in carotenoid proportions during six tuberous root developmental stages of sweetpotatoes with three flesh colors.

**Additional file 2. Fig. S2**: Amino acid sequence alignment of the IbCCD4 with CCD4s from nine other plant species. Blue asterisks indicate the four highly conserved histidine residues acting as iron-ligating cofactors; blue asterisks indicate the conserved glutamates or aspartate required for fixing the iron-ligating histidine residues. The GenBank accession numbers of the amino acid sequences used included the following: ItCCD4 (*Ipomoea triloba*, XP_031124907.1), InCCD4 (*Ipomoea nil*, XP_019156361.1), StCCD4 (*Solanum tuberosum*, XP_006359966.1), SlCCD4 (*Solanum lycopersicum*, XP_004246004.1), VvCCD4 (*Vitis vinifera*, AGT63321.1), OfCCD4 (*Osmanthus fragrans*, ABY60887.1), DmCCD4 (*Dendranthema morifolium*, BAF36656.2), PpCCD4 (*Prunus persica*, PRUPE_1G255500), and AtCCD4 (*Arabidopsis thaliana*, AT4G19170).

**Additional file 3. Fig. S3**: A phylogenetic tree of IbCCD4 and CCD4s from nine other plant species. The scale bar indicates nucleotide substitutions per site.

**Additional file 4. File S1**: Detailed method of metabolomic analysis of carotenoids by UHPLC-APCI-MS/MS. **File S2**: The potential binding motifs of IbCBF2 and IbWRKY20 in the 260-bp promoter sequence of IbCCD4.

**Additional file 5. Table S1**: Primers used in this study. **Table S2**: Determination of the carotenoid contents during six tuberous root developmental stages of sweetpotatoes with three flesh colors by spectrophotometry (A454/g). **Table S3**: Determination of carotenoid compositions and contents during six tuberous root developmental stages of sweetpotatoes with three flesh colors by UHPLC-APCI-MS/MS (μg/g DW). **Table S4**: Relative expression levels of thirteen genes involved in carotenoid metabolic pathway. **Table S5**: Determination of carotenoid compositions and contents in tuberous roots of *IbCCD4*-overexpressing lines and non-transgenic sweetpotato by UHPLC-APCI-MS/MS (μg/g DW). **Table S6**: *Cis*-acting element locate in the *IbCCD4* promoter.

## Data Availability

All data generated or analyzed during this study are included in this published article and its Supplementary files.

## Abbreviations

GGPS: Geranylgeranyl diphosphate synthase
PSY: Phytoene synthase
PDS: Phytoene desaturase
ZISO: ζ-Carotene isomerase
ZDS: ζ-Carotene desaturase
CRTISO: Carotene isomerase
LCYB: Lycopene β-cyclase
LCYE: Lycopene Ɛ-cyclase
CHYB: β-Carotene hydroxylase
CHYE: Ɛ-Carotene hydroxylase
ZEP: Zeaxanthin epoxidase
NSY: Neoxanthin synthase
CCD1: Carotenoid cleavage dioxygenase 1
CCD4: Carotenoid cleavage dioxygenase 4
NCED: 9-*cis*-Epoxycarotenoid dioxygenase
IPP: Isopentenyl diphosphate
DMAPP: Dimethylallyl diphosphate
GGPP: Geranylgeranyl diphosphate
ABA: Abscisic acid
SL: Monochromatic gold lactone
UHPLC-APCI-MS/MS: High-performance liquid chromatography-tandem mass spectrometry
RT-qPCR: Quantitative real-time polymerase chain reaction
