# Supplementary figures and images for: Integrated metabolic and transcriptional analysis reveals the role of carotenoid cleavage dioxygenase 4 (IbCCD4) in carotenoid accumulation in sweetpotato tuberous roots
Source: Biotechnol Biofuels Bioprod. 2023 Mar 14;16:45. doi: 10.1186/s13068-023-02299-y (PMC10012543; doi:10.1186/s13068-023-02299-y)

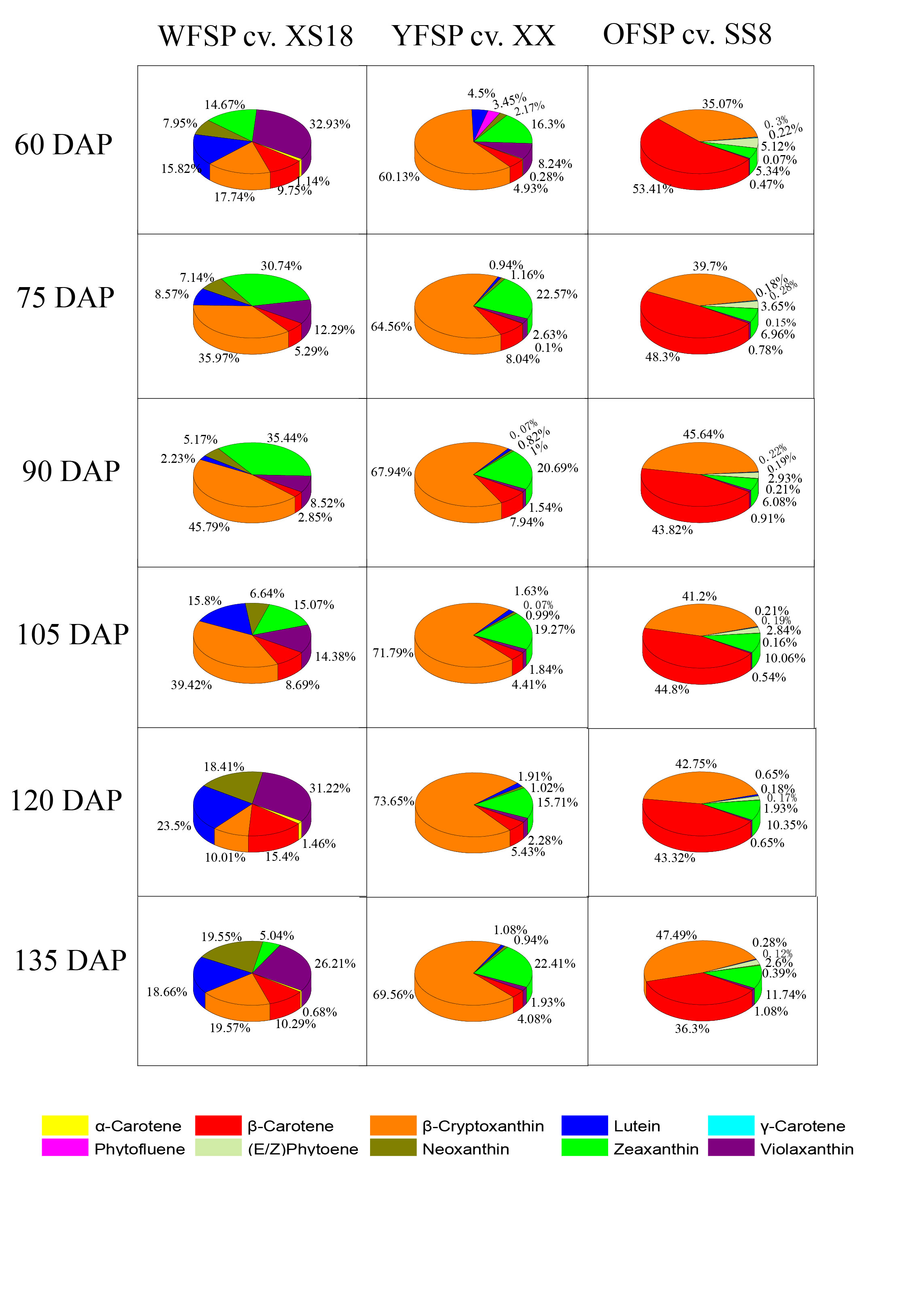

Supplement: Supplementary file 1 — Additional file 1. Fig. S1: Dynamic changes in carotenoid proportions during six tuberous root developmental stages of sweetpotatoes with three flesh colors. [file 13068_2023_2299_MOESM1_ESM.jpg]

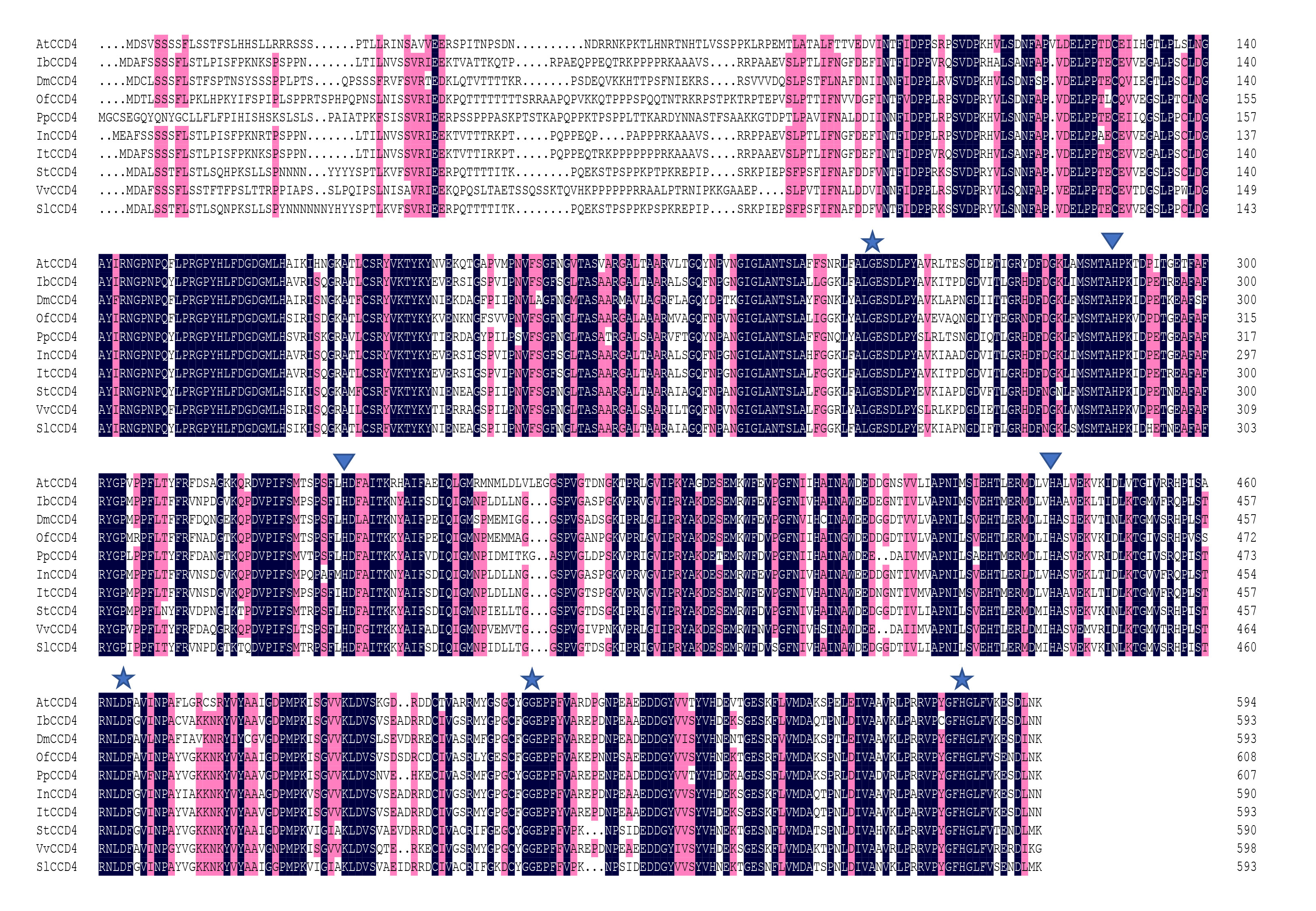

Supplement: Supplementary file 2 — Additional file 2. Fig. S2: Amino acid sequence alignment of the IbCCD4 with CCD4s from nine other plant species. Blue asterisks indicate the four highly conserved histidine residues acting as iron-ligating cofactors; blue asterisks indicate the conserved glutamates or aspartate required for fixing the iron-ligating histidine residues. The GenBank accession numbers of the amino acid sequences used included the following: ItCCD4 (Ipomoea triloba, XP_031124907.1), InCCD4 (Ipomoea nil, XP_019156361.1), StCCD4 (Solanum tuberosum, XP_006359966.1), SlCCD4 (Solanum lycopersicum, XP_004246004.1), VvCCD4 (Vitis vinifera, AGT63321.1), OfCCD4 (Osmanthus fragrans, ABY60887.1), DmCCD4 (Dendranthema morifolium, BAF36656.2), PpCCD4 (Prunus persica, PRUPE_1G255500), and AtCCD4 (Arabidopsis thaliana, AT4G19170). [file 13068_2023_2299_MOESM2_ESM.jpg]

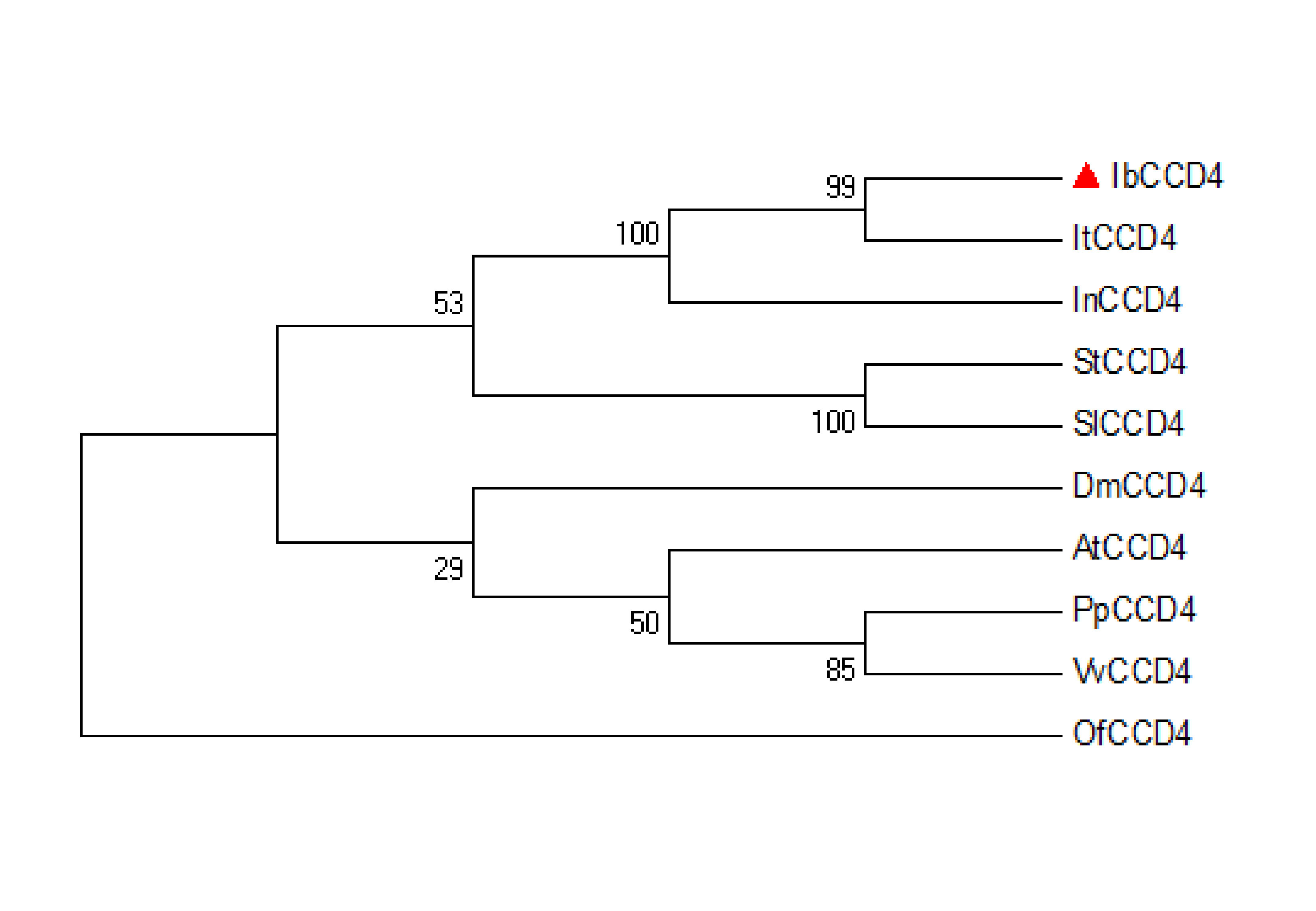

Supplement: Supplementary file 3 — Additional file 3. Fig. S3: A phylogenetic tree of IbCCD4 and CCD4s from nine other plant species. The scale bar indicates nucleotide substitutions per site. [file 13068_2023_2299_MOESM3_ESM.jpg]
