# Supplementary material for: Integrated metabolic and transcriptional analysis reveals the role of carotenoid cleavage dioxygenase 4 (IbCCD4) in carotenoid accumulation in sweetpotato tuberous roots
Source: Biotechnol Biofuels Bioprod. 2023 Mar 14;16:45. doi: 10.1186/s13068-023-02299-y (PMC10012543; doi:10.1186/s13068-023-02299-y)
Supplement: Supplementary file 4 — Additional file 4. File S1: Detailed method of metabolomic analysis of carotenoids by UHPLC-APCI-MS/MS. File S2: The potential binding motifs of IbCBF2 and IbWRKY20 in the 260-bp promoter sequence of IbCCD4. [file 13068_2023_2299_MOESM4_ESM.docx]

**Supplementary File S1.**

**The detailed method of metabolomic analysis of carotenoids by UHPLC-APCI-MS/MS.**

The extraction steps were as follows: (1) The freeze-dried tuberous root samples were ground to a powder with a grinder (MM 400, Retsch, Haan, Germany). (2) A mixture of n-hexane: acetone: ethanol was prepared as the extraction solution, and then 0.01% BHT (g/mL) and 50 mg of ground powder were mixed with an appropriate amount of the extraction solution and an internal standard (Sigma). (3) The mixture was vortexed at room temperature for 20 min, then centrifuged, and the supernatant was removed. (4) Steps (2) and (3) were repeated, and the supernatants were combined and evaporated to dryness. (5) The sample was resuspended with an appropriate amount the solution, a mixture of methanol and methyl tert-butyl ether (MTBE). (6) After filtering through a 0.22 μm membrane, the samples were analyzed using an LC-atmospheric pressure chemical ionization (APCI)-MS/MS system (UHPLC ExionLC AD; MS Applied Biosystems 6500 Triple Quadrupole) (Metware Biotechnology Co., Ltd, Wuhan, China).

All metabolites were annotated by MetWare database. The HPLC analytical conditions were as follows: column, YMC C30 (3 µm, 2 mm × 100 mm); solvent system, methanol: acetonitrile (3:1, V/V) (0.01% 2, 6-Ditert-butyl-4-methylphenol (BHT), 0.1% formic acid) and MTBE (0.01% BHT); gradient program, 100:0 V/V at 0 min, 100:0 V/V at 3 min, 58:42 V/V at 6 min, 20:80 V/V at 8 min, 5:95 V/V at 9 min, 100:0 V/V at 9.1 min, and 100:0 V/V at 11 min; flow rate, 0.8 mL/min; temperature, 28 °C; injection volume, 2 μL. The API 6500 Q TRAP LC/MS/MS System, equipped with an APCI Turbo Ion-Spray interface, was operated in positive ion mode and controlled by Analyst 1.6.3 software (AB Sciex). The APCI source operation parameters were as follows: ion source, APCI^+^; source temperature, 350 °C; curtain gas (CUR), 25.0 psi; and collision gas (CAD), medium. Declustering potential (DP) and collision energy (CE) for individual multiple reaction monitoring (MRM) transitions were performed with further DP and CE optimization. A specific set of MRM transitions was monitored for each period according to the carotenoids eluted within each period. All metabolites were quantified using MRM. The integrated peak area of each carotenoid detected in the samples was substituted into the linear equations of standard curves for content calculation; finally, the absolute content data for the carotenoids in the actual samples was obtained.

Carotenoid content (μg/g) =A* V*10^-3^/W, where A is the concentration (μg/mL) obtained by substituting the integrated peak area of a carotenoid in the sample into the corresponding standard curve, V is the resuspension volume (μL), and W is the mass of the weighed sample (g).

**Supplementary File S2.**

The potential binding motifs of IbCBF2 and IbWRKY20 in the 260-bp promoter sequence of *IbCCD4.*

GACTTTTAGATATAAGAGTCATGTTCCACTCGGTTTTGGCTACCCTTTTAGATATAAGAACCATGTTTGATCCACTTGGCGTTGGCTACCGGAAGAGTAGATATTAAACTTGAAGTCTGTGTCTTATTTTGTCACATATGAAACGTGTATATATGTAGAAGCTGGTTAGTTGTAGCCATCCATTACATAATTAAATAGAGAAGGAAACCTGTAAGTTAAATATATTTCATACAAAATGTGAGCCTCACGTAGTTTGTGAT

Note:

CBF binding motif: A/GCCGAC

two potential sites (with a single nucleotide change, in lower case) present in the promoter in reverse orientation: cTCGGT, GTtGGC (yellow)

WRKY binding motif: W-box: TTGACY (Y: C or T); core motif TGAC

one potential site (with a single nucleotide change) present in the promoter in reverse orientation: AGTCAt (blue)

In addition, it contains T/G-box and ACGT (purple) core motifs: potential binding motif for bZIPs and bHLH.

ABRE element: ACGTG(green)
